# Age-related differences in white matter diffusion measures in autism spectrum condition

**DOI:** 10.1186/s13229-020-00325-6

**Published:** 2020-05-18

**Authors:** Abigail Thompson, Asal Shahidiani, Anne Fritz, Jonathan O’Muircheartaigh, Lindsay Walker, Vera D’Almeida, Clodagh Murphy, Eileen Daly, Declan Murphy, Steve Williams, Sean Deoni, Christine Ecker

**Affiliations:** 1 Department of Forensic & Neurodevelopmental Sciences, Institute of Psychiatry, Psychology & Neuroscience, King’s College London, London, UK; 2 Developmental Change & Plasticity Lab, Department of Psychology & Language Sciences, University College London, 26 Bedford Way, Bloomsbury, London, WC1H 0AP UK; 3 The Centre for Research in Autism and Education (CRAE), Psychology and Human Development, UCL, London, UK; 4 Centre for the Developing Brain, Department of Perinatal Imaging and Health, St. Thomas’ Hospital, King’s College London, London, UK; 5 Department of Neuroimaging, Institute of Psychiatry, Psychology & Neuroscience, King’s College London, London, UK; 6 MRC Centre for Neurodevelopmental Disorders, King’s College London, London, UK; 7 Advanced Baby Imaging Lab, Hasbro Childrens Hospital, Providence, RI USA; 8 Pediatrics and Radiology, Warren Alpert medical school, Brown University, Providence, USA; 9 Maternal, Newborn & Child Health Discovery & Tools at the Bill and Melinda Gates Foundation, Seattle, USA; 10 Department of Child and Adolescent Psychiatry, Psychosomatics and Psychotherapy, University Hospital, Goethe University Frankfurt am Main, Deutschordenstrasse 50, 60528 Frankfurt am Main, Germany

**Keywords:** Autism, Tract-based spatial statistics, Diffusion weighted imaging, Connectivity

## Abstract

**Background:**

Autism spectrum condition (ASC) is accompanied by developmental differences in brain anatomy and connectivity. White matter differences in ASC have been widely studied with diffusion imaging but results are heterogeneous and vary across the age range of study participants and varying methodological approaches. To characterize the neurodevelopmental trajectory of white matter maturation, it is necessary to examine a broad age range of individuals on the autism spectrum and typically developing controls, and investigate age × group interactions.

**Methods:**

Here, we employed a spatially unbiased tract-based spatial statistics (TBSS) approach to examine age-related differences in white matter connectivity in a sample of 41 individuals with ASC, and 41 matched controls between 7–17 years of age.

**Results:**

We found significant age-related differences between the ASC and control group in widespread brain regions. This included age-related differences in the uncinate fasciculus, corticospinal tract, inferior longitudinal fasciculus, inferior fronto-occipital fasciculus, anterior thalamic radiation, superior longitudinal fasciculus and forceps major. Measures of fractional anisotropy (FA) were significantly positively associated with age in both groups. However, this relationship was significantly stronger in the ASC group relative to controls. Measures of radial diffusivity (RD) were significantly negatively associated with age in both groups, but this relationship was significantly stronger in the ASC group relative to controls.

**Limitations:**

The generalisability of our findings is limited by the restriction of the sample to right-handed males with an IQ > 70. Furthermore, a longitudinal design would be required to fully investigate maturational processes across this age group.

**Conclusions:**

Taken together, our findings suggest that autistic males have an altered trajectory of white matter maturation relative to controls. Future longitudinal analyses are required to further characterize the extent and time course of these differences.

## Introduction

Autism spectrum condition (ASC) is a life-long neurodevelopmental condition characterized by atypical social communication and reciprocity and a propensity for repetitive and stereotyped behaviour [1]. These principal symptoms typically manifest before the age of 2 years and are accompanied by developmental differences in brain anatomy and connectivity [2]. The neural systems underlying ASC are complex and involve alterations in multiple, spatially distributed neurocognitive systems [3]. Thus, our understanding of the neuroanatomy of ASC remains incomplete.

Despite the large phenotypic heterogeneity and complex aetiology of ASC, it is well accepted that autistic individuals show an atypical trajectory of brain development. For example, neurodevelopmental differences in brain maturation have been observed on the global level, and age-related differences in overall brain growth of total grey and white matter volume have been reported [4, 5]. More specifically, it has been suggested that individuals with ASC undergo a period of accelerated brain growth during early postnatal life, causing the brain to be larger in early childhood relative to typically developing controls [6]. The precocious increase in total brain volume is followed by a period of atypically slow or arrested growth throughout the remainder of childhood, so that no global differences are generally observed by adulthood [5]. Increased white matter development has been suggested to contribute to early brain overgrowth in childhood [7], suggesting that brain maturation in ASC also affects the development of brain ‘connectivity’.

There is evidence from structural and functional magnetic resonance imaging, positron emission tomography, and electroencephalography studies that ASC is associated with altered brain connectivity [8–11]. Atypical brain functional connectivity has been documented in both adults [12] and children with ASC [8, 13] and particularly in the neural networks that mediate autistic symptoms and traits. For example, altered functional connectivity of temporal-lobe networks [14] has been associated with social/emotional behaviours, and abnormalities in fronto-striatal networks have been associated with ritualistic/repetitive behaviours in ASC. There is also evidence of atypical white matter structural connectivity between these regions in ASC, which has been extensively studied using diffusion tensor imaging (DTI) [15].

Diffusion imaging studies report widespread differences in white matter microstructure among infants [16], children and adolescents [17, 18], and adults on the autism spectrum [11, 19]. More specifically, increased mean fractional anisotropy (FA) has been reported in autistic infants and toddlers [20, 21], which may indicate increased tract coherence and axonal alignment in the condition. However, this pattern seems to be reversed (i.e. lower FA values in ASC) in childhood and adolescence [22]. Some studies have also observed the opposite, with decreased FA in toddlers [23] and increased FA in later childhood [24, 25], whilst some studies have found no significant group differences in FA in children and adolescents at all [26]. Fewer differences in FA diffusion measures have been described in later adolescence and adulthood [27, 28], suggesting that between-group differences in white matter microstructure might disappear with increasing age.

Differences have also been reported in mean diffusivity (MD) and radial diffusivity (RD), but there is some disagreement. MD indicates the degree of direction-independent average diffusivity and has been shown to decrease over the course of healthy white matter maturation [29]. Studies have mainly reported no difference or higher MD values in individuals with ASC relative to controls [30, 31], although lower MD values have also been reported in one study [32]. Last, there are reports of increases in RD in both children [33] and adults with ASC [19], which may reflect changes in underlying white matter properties including reduced myelination [34].

Taken together, these findings contribute to a heterogeneous body of literature regarding diffusion imaging and ASC. This heterogeneity may indicate that the pattern of white matter abnormality varies across the investigated age range of participants and affects widespread neural systems rather than isolated brain regions. Moreover, differences in the employed methodology may affect the results. For example, the majority of DTI studies that have previously been conducted in ASC are based on a region of interest (ROI) approach [35]. ROI approaches rely on *a priori* hypotheses regarding the specific white matter tract or region under investigation. As it is well established that ASC is related to diffuse and spatially distributed white matter differences [3], an exploratory whole-brain approach such as TBSS is particularly well suited for examining this group of individuals.

Some studies have previously used TBSS to investigate ASC but interpretation of findings is hindered by methodological limitations and varying methodological approaches across studies. On the whole, TBSS studies have been a small scale, including a narrow age range of participants or a heterogeneous sample (including both right- and left-handed participants) [36, 37]. TBSS studies that have included the largest samples have most frequently included adults [19, 37, 38]. Studies with large sample sizes that have included children and adolescents have primarily included mixed genders [39–42], which may contribute to discrepancies across studies, due to the finding that autistic males and females may have distinct white matter developmental profiles [43].

Technical factors also vary across studies and may affect the results reported. Calculation of diffusion imaging parameters is affected by artefacts, which can be caused by subject-related factors such as participant motion. Another potential source of artefacts in diffusion imaging is cardiac pulsation [44]. The vast majority of studies do not report correction for this [21, 43, 45–50], suggesting that this important methodological factor has, on the whole, been overlooked. Finally, after corrections have been made, it is necessary to rotate the b-matrix to ensure accuracy of diffusion imaging parameters. Similar to cardiac gating, b-matrix rotation is largely not reported within studies [45, 47, 48, 51], suggesting this may be a contributor to the contradictory results reported across studies.

One study that did take a highly rigorous methodological approach reported reductions in FA in the autism group and a stronger developmental trajectory in the ASC group. This study included a large sample of male, right-handed ASC participants and controls who were matched for IQ (aged 10–24), corrected for motion and cardiac pulsation and rotated the b-matrix [52]. This potentially points towards a particular developmental trajectory, in which white matter development ‘normalises’ by adulthood in the autism group. However, as this is only one study of many that has taken such a rigorous methodological approach, more studies are needed to conclusively characterise the developmental trajectory of white matter in autism. Additionally, this study corrected for cardiac pulsation during pre-processing. A more direct approach is to use cardiac gating during acquisition. We are not aware of any TBSS study that has used cardiac gating during acquisition. Furthermore, the age range of participants included in this particular study includes the transition from adolescence to young adulthood, which is itself associated with distinct neurodevelopmental patterns [53]. Given the neurodevelopmental nature of autism, it is important for robustly designed studies with large sample sizes to focus on earlier developmental time points.

## Materials and methods

### Participants

Forty-one males with ASC (aged 7–17 years) and forty-one typically developing male controls (aged 8–17 years) were recruited by advertisement and assessed at the Centre for Neuroimaging Sciences, King’s College London, The Institute of Psychiatry, Psychology & Neuroscience, London. All participants were right-handed (measured using The Edinburgh Handedness inventory) [54] native English speakers and all had an IQ > 70. Exclusion criteria included pre-existing medical conditions or complications (e.g. head trauma, epilepsy), use of medication affecting brain function, a history of major psychiatric disorder (e.g. psychosis), chromosomal abnormality (e.g. fragile X, Tuberous Sclerosis, 22q11.2 deletion syndrome), and any MRI contraindications. Intellectual ability was assessed using the WASI [55]. For the autistic group, inclusion was based on an expert clinical diagnosis of autism using the International Statistical Classification of Diseases, 10th Revision (ICD-10) research criteria and confirmed using the Autism Diagnostic Interview-Revised (ADI-R) [56] (all cases reached ADI-R algorithm cutoff for autism on the domains of impaired reciprocal social interaction, communication, and repetitive behaviours and stereotyped patterns, although failure to reach cutoff for autism in a single domain by 1 point was permitted). Current symptoms were assessed using the Autism Diagnostic Observation Schedule [57], but were not used as an inclusion criterion. All participants and their parents or guardians gave informed written consent/assent in accordance with the ethics approval by the National Research Ethics Committee, Suffolk, UK.

### MRI

All imaging for this study was acquired on a General Electric 3 T MR system. Diffusion tensor MRI scans were acquired with a spin-echo echo-planar imaging (SE-EPI) double refocused sequence providing a whole head coverage with isotropic image resolution (2.4 × 2.4 × 2.4 mm); 32 diffusion-weighted volumes with different non-collinear diffusion directions with b-factor 1300 sec/mm^2^ and 6 non-diffusion-weighted volumes with 60 slices, no slice gap, TE 104.5 ms, TR 20 R-R intervals, 128 × 128 acquisition matrix and FOV = 30.7 cm^2^ were peripherally gated (parameters compatible with [58]). Total acquisition time was approximately 12 min. If participants were unable to tolerate scanning or obvious head movement was detected during the acquisition (due to anxiety or hyperactivity for example), they were invited to return for a second time—at which time scan quality was usually significantly improved.

### Preparation of data

All data were initially visually inspected by raters at the IOPPN, KCL UK, and Brown School of Engineering, Rhode Island, to ensure inter-rater reliability. The data then went through a comprehensive correction pipeline using the TORTOISE software (https://science.nichd.nih.gov/confluence/display/nihpd/TORTOISE) [59], which registers the volumes of a DTI dataset to reduce the effects of motion and eddy current-based deformations. Corrections were performed in the native space of each subject, and appropriate rotations were applied to the b-matrix [60]. All deformations in TORTOISE were computed and applied in a single step to avoid multiple interpolations of the data.

After correction, expert raters manually inspected the data and removed any volumes with residual artefacts. A total of ten volumes (across seven subjects) were removed, accounting for 0.3% of the total number of volumes acquired across the cohort. Differences in subject motion have been shown to be an important consideration for group comparisons of DTI data [61]. Therefore, to rule out the effect of age and between-group differences in head motion, a univariate analysis of variance was carried out to identify the main effect of age and group, as well as their interaction on the number of volumes removed and the amount of distortion and motion-correction applied during processing. For this purpose, transformation data detailing the degree of distortion and motion correction for each participant was extracted using the TORTOISE software.

### Estimation of the diffusion tensor

Following the completion of quality control procedures, all subsequent analysis was carried out using the FMRIB Software Library (FSL, www.fmrib.ox.ac.uk/fsl). First, skull and non-brain tissue were removed using BET. Voxel-wise values of FA, MD and RD were then calculated.

### Voxel-wise analysis

Voxel-wise statistical analysis was performed on the FA, MD and RD data using TBSS [62]. TBSS tests for between-group differences in diffusion measures across a ‘skeleton’ of WM tracts and across the whole brain. This procedure includes a number of steps. First, individual FA maps were non-linearly aligned to a standard space using a target image. In this study, the target image was chosen to be the most representative FA image, using the flag designation ‘-n’. This is the recommended option for studies of adolescents and young children. The most representative FA image (the target) was from a member of the ASC group, aged 13.5 years. After image registration, a cross-subject mean FA image was calculated, which informed the generation of the WM tract ‘skeleton’, thresholded at FA > 0.3 to include major WM pathways whilst excluding peripheral tracts that are more vulnerable to partial volume effects and/or inter-subject variability. Finally, each subject’s FA, MD, and RD values were projected onto the group skeleton, and the resulting data was fed into voxel-wise analysis. For statistical analysis, we used the randomize function within FSL to conduct permutation-based nonparametric statistics [63] with 10,000 permutations. Areas of significant difference were displayed as a *P* value image, where *P* < 0.05, corrected for multiple comparisons across space via threshold-free cluster enhancement [64].

In order to establish the most parsimonious model of age-related differences, we initially examined linear and quadric effects with regards to age-related differences in diffusion measures. Applying a quadratic age term did not significantly increase the goodness of fit, the more parsimonious linear model was thus favoured for examining WM integrity. The GLM used in the present study therefore included a main effect of group, a linear term for age, as well as the interaction between age and group.

### Correlation with symptom measures

Finally, to investigate the relationship with the severity of autistic symptom and diffusion measures, correlation analyses were conducted between the diffusion maps and ASC severity measured by the ADI-R and the ADOS. For all voxel-wise analyses, affected white matter structures were identified with the John Hopkins University white matter atlas [65].

## Results

### Participants

Participant demographics are listed in Table 1. Groups did not differ significantly in either age (*t* (80) = 0.49, *p* = 0.63) or IQ (*t* (80) = − 0.30, *p* = 0.76).

### Movement

There was no significant effect of age (*f* (48) = 0.91, *p* = 0.61), group (*f* (1) = 1.07, *p* = 0.32), or group × age interaction (*f* (17) = 1.96, *p* = 0.09) on the number of volumes removed during quality control. No significant group differences were found in the application of distortion and motion correction (*t* (82) = − 1.72, *p* = 0.09). And no significant effect of age (*f* (48) = 1.76, *p* = 0.12), group (*f* (1) = 0.87, *p* = 0.37), or group × age interaction (*f* (17) = 1.96, *p* = 0.08) on the degree of distortion and motion correction applied during processing.

### TBSS analysis

**Fig. 1:**
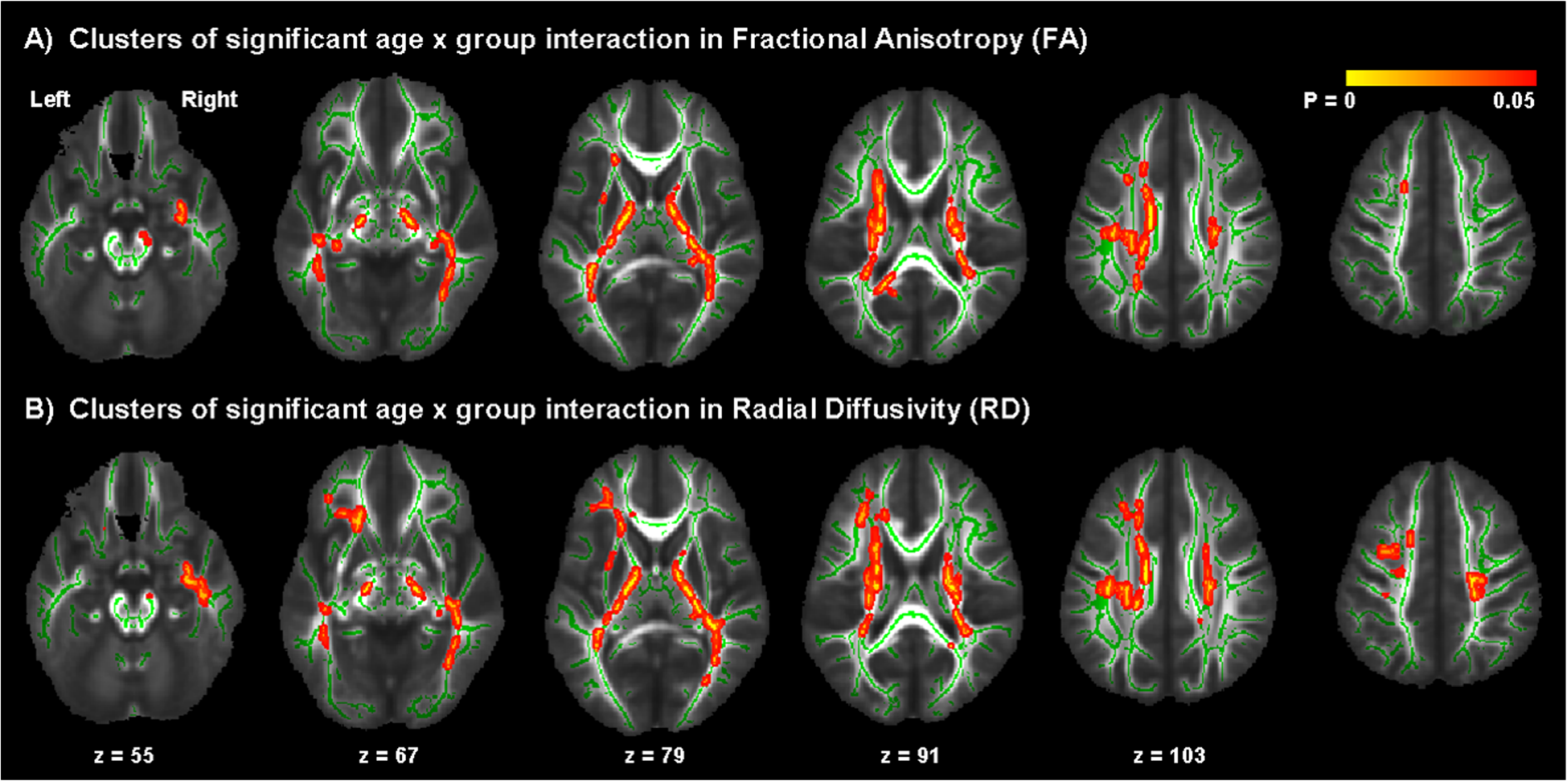
Results of TBSS analysis of FA and RD. Age × group interaction for **a** FA and **b** RD, overlaid mean FA computed from all subjects. FA is positively associated with age in both groups but this association is stronger in the autism spectrum condition (ASC) group than in typical controls, whereas RD is negatively associated with age in both groups but this association is stronger in the ASC group than in the control group. Statistically significant voxels are displayed in red-yellow; white matter skeleton voxels are displayed in green, overlaid onto mean FA computed from all subjects. Significance was set at *p* < 0.05 and was corrected for multiple comparisons with family wise error. Group differences ‘thickened’ and images flipped in the right-left plane for visualization purposes

The TBSS analysis did not reveal any significant between-group differences in FA, RD or MD. There were, however, significant age × group interactions for both measures of FA and RD in the UF, corticospinal tract, ILF, IFOF, anterior thalamic radiation, SLF and forceps major (Fig. 1). In these regions we found that measures of FA were significantly positively associated with age in both groups; however, this relationship was stronger in the ASC group in comparison to controls. Measures of RD were negatively associated with age in both groups; however, this relationship was stronger in the ASC group in these ROIs. The age × group interaction for measures of MD approached statistical significance, but did not reach the statistical threshold of *p* < 0.05.

**Table 1.** Subject demographics. Data expressed as mean ± standard deviation (range). There were no significant between-group differences in age (*t* (80) = 0.49, *p* = 0.63) or IQ (*t* (80) = − 0.30, *p* = 0.76)

	**ASC (*n*= 41)**	**Control (*n*= 41)**
Age (years)	13.1 ± 2.7 (7–17)	12.9 ± 2.5 (8–17)
IQ (WASI)	110.1 ± 15.4 (70–140)	110.9 ± 11.3 (79–132)
ADI-R reciprocal social interaction	17.7 ± 4.4 (10–26)	–
ADI-R communication	16.3 ± 3.8 (8–23)	–
ADI-R restricted, repetitive, stereotyped behaviour	5.5 ± 2.3 (2–11)	–
ADOS communication	3.7 ± 1.5 (1–6)	–
ADOS reciprocal social interaction	7.4 ± 2.7 (3–13)	–
ADOS imagination/creativity	0.9 ± 0.7 (0–2)	–
ADOS Stereotyped behaviours, restricted interests	1.5 ± 1.5 (0–6)	–

**Fig. 2:**
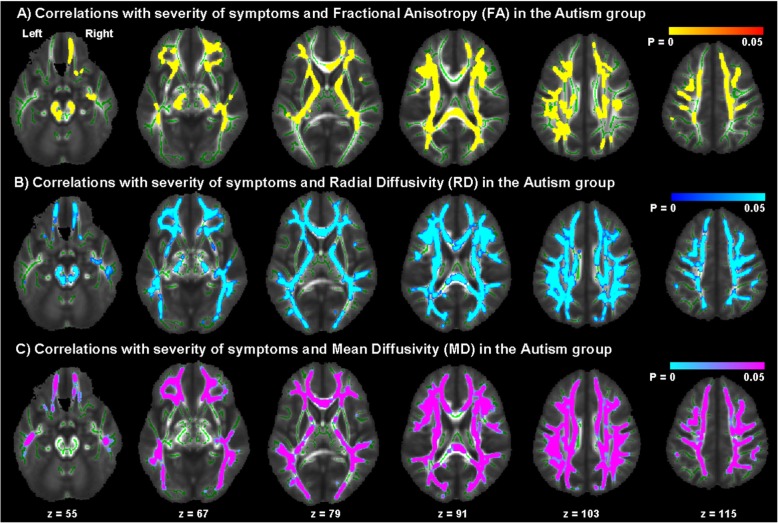
Correlations with ADOS-D symptom measures. Clusters of significant correlation between the ADOS-D measures in the ASC group and **a** FA, **b** RD and **c** MD. Correlations were negative for FA and positive for RD and MD. Statistically significant voxels are displayed in red, blue and purple; white matter skeleton voxels are displayed in green. Significance was set at *p* < 0.05 and was corrected for multiple comparisons with family wise error. Group differences ‘thickened’ and images flipped in the right-left plane for visualization purposes

There were significant correlations between measures of symptom severity based on the ADOS domain of ‘Stereotyped Behaviours and Restricted Interests’ in spatially distributed brain regions including the UF, ILF, IFOF, corticospinal tract, and the anterior thalamic radiation (Fig. 2). There were no significant correlations with the other ADI or ADOS domains. These regions also overlap significantly with the brain regions where we observed a significant age × group interaction in the TBSS.

**Fig. 3:**
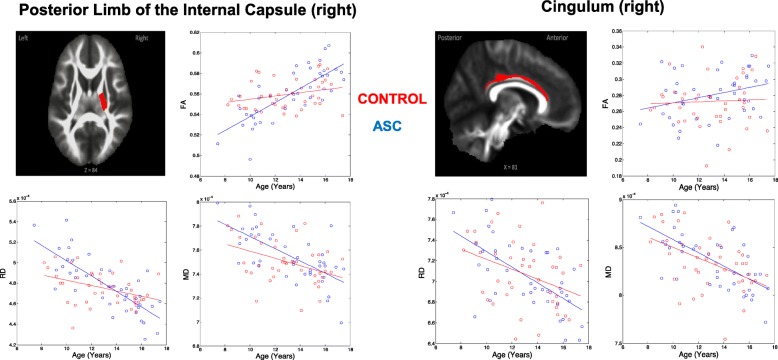
Examples of age interactions in regions of interest. Examples of two regions of interest are shown for illustrative purposes (all sixteen regions of interest are included in supplementary figure 1). Regions derived from the John Hopkins University white matter atlas are co-registered with mean diffusion data. For each white matter pathway, a plot of the raw data and mean linear trend-line can be seen for FA, MD and RD. Blue corresponds to participants with ASC and red with typically-developing controls. Images flipped in the right-left plane for visualization purposes

In order to visualise the nature of the interactions across different white matter regions, we applied region of interest masks with 16 WM regions/pathways from the John Hopkins University white matter atlas, from which diffusion measures were extracted. The masks were co-registered with the diffusion data, and mean FA, MD and RD values were obtained for each participant in each of the ROIs. Scatter plots of raw data with superimposed mean linear development trajectories for three of the sixteen WM pathways we examined are displayed in Fig. 3, for scatter plots of all 16 WM pathways refer to Supplementary Figure, 1. These are presented for illustration purposes only.

## Discussion

We found significant differences in the trajectory of white matter development between male children with ASC and typically developing controls, as indicated by group differences in the relationship between diffusion measures and age. The developmental differences in white matter were observed in spatially distributed white matter regions across the brain. In both groups, measures of FA were significantly positively associated with age, while measures of both RD and MD were significantly negatively associated with age. These findings are in agreement with previous neuroimaging studies suggesting that neurodevelopmental changes in diffusivity accompany the general maturation of white matter connections in the brain [29]. We further observed that males with ASC showed a stronger positive relationship between FA with age, which was accompanied by a concomitant negative relationship with MD and RD, particularly in brain regions and white matter tracts that have previously been implicated in the disorder. Additionally, we found that these DTI measures significantly correlated with the severity of stereotyped behaviours and restricted interests as measured by the ADOS (domain D) within the ASC group. On the whole, our findings corroborate previous studies that have noted widespread white matter differences in multiple brain areas in ASC and explicitly highlight the importance of consideration of age in brain development and behaviours for autistic males.

Our findings of extensive age-related between-group differences across spatially distributed white matter tracts are consistent with studies that report stronger age-related development in autism [47, 48, 50, 52, 66]. Some studies, however, have reported the opposite pattern, that the relationship with age is stronger in controls and smaller or absent in the ASD group [21, 36, 37, 49, 67]. These contrasting findings may relate to a number of methodological differences relating to participant inclusion criteria, such as inclusion of both genders [36, 37, 47, 48, 67] and mixed handedness [36, 37] as well as small sample sizes [21, 36, 47–49, 67]. Technical factors may also have contributed to contrasting results in previous studies, such as failing to correct for motion, rotate the b-matrix [21, 36, 37, 43, 47–49, 67], or account for cardiac pulsation [45, 47, 48, 51]. We note that only one previous study took the important methodological steps of b-matrix rotation and accounting for cardiac pulsation in the context of TBSS [52] and all the others did not. In contrast to this study, we used cardiac gating during acquisition. We are not aware of any other TBSS study of autism that has done this. The age-related differences reported by this study [52] are in line with ours, with a stronger relationship in the autism group, in an older sample (aged 10–24). Our findings therefore extend the findings of Fitzgerald to a younger age group, in a robust and well-controlled study, pointing towards an altered developmental trajectory of white matter across late childhood and adolescence in autistic males.

The adaptive implications of these differences in the white matter developmental trajectory are not fully established. Some studies suggest there is ‘normalisation’ of white matter indices into adulthood, which may be associated with improvements of functioning [5, 52]; however, other studies report that reduced connectivity indices are associated with impairments in autistic adults [11]. In the present study, we found that lower stereotyped behaviour ADOS scores were positively associated with FA and negatively associated with RD and MD values. This trend was observed across widespread brain areas. This pattern of correlations suggests that whilst the ASC group is characterized by a stronger positive association between in FA and age, this is concomitant with a reduction of ASC symptomatology. Thus, the accelerated change in diffusion measures in the ASC group may in fact be adaptive.

Whilst we have reported extensive age-related between-group differences at both the voxel- and region-wise levels, we did not observe any significant between-group differences per se. This could indicate that one of the most important effects, aging, is overlooked in simple group comparisons. Some studies investigating a similar age range to ours have, in fact, reported between-group differences [68–71]; however, these have all included both males and females. As there are reported sex differences both in terms of white matter development per se [72], and in terms of white matter development in the context of autism [43, 73, 74], the discrepancy here may relate to the inclusion of both sexes. We note that in other studies across similar age ranges with exclusively male participants no between-group differences have also been reported (e.g. [75]), suggesting that an absence of between-group differences in males may be a consistent finding between childhood and adolescence.

It has been proposed that autism is associated with a unique trajectory of brain connectivity maturation, characterised by ‘over’ connectivity very early in childhood [76, 77], which then reverses over development. This leads to either ‘normalisation’ or, alternatively, reduced measures of white matter indices in ASC cohorts in adulthood (which explains why some studies, with all-male cohorts, including older ages report reduced measures of connectivity, e.g. [52]). If such a developmental trajectory does characterise autism, there must be a crossover point between early childhood and adulthood, at which diffusion indices between autistic individuals and controls would intersect, leading to no identifiable between-group differences. It may be the case that mid-late childhood and adolescence, as studied in the present cohort, constitutes such a developmental time point for ASC males. Ultimately, in a cross-sectional study such as this, we are not able to disentangle such effects. Future longitudinal studies will be required.

Overall, diffusion measures reflect a number of underlying biological processes, which need to be considered when interpreting the current findings. Alterations in FA, RD and MD may suggest differences in fibre organization and geometry, myelin formation, and myelin remodelling, as well as inflammation and gliosis [34, 78, 79]. This lack of specificity of diffusion measures constitutes an inherent limitation of DTI. This is further compounded by the finding that DTI measures may also be impacted by extrinsic factors that render group-differences specious. An example of such an effect comes from group differences in head motion in the scanner. Increased in-scanner motion in one group can lead to significantly decreased FA and increased RD in comparison to a relatively motionless control group [61]. These findings are particularly salient when investigating children and individuals with ASC, who may display increased movement inside the scanner. Our results, however, cannot be explained by this as the amount of in-scanner motion was not found to be different between the control and ASC groups. In line with this, we did not find decreased FA and increased RD in the group comparison, which would have been predicted as a result of increased motion [61].

## Limitations

The generalisability of our findings is limited by the restriction of the sample to right-handed males with an IQ > 70. Thus, it is not possible to determine the applicability of these findings to autistic females, or individuals with an IQ of < 70, which constitutes a substantial proportion of autistic individuals. Furthermore, the cross-sectional nature of the study means that the increased heterogeneity associated with ASC cannot be accounted for and also limits our understanding of maturational processes across this age group, for which a future study using a longitudinal design would be required.

## Conclusions

To conclude, using a rigorous methodological approach, our findings indicate a distinct neurodevelopmental trajectory of white matter development in young males with ASC. However, without further histological validation, these differences cannot be attributed to a specific biological process or feature. As the differences we see in FA, MD and RD are widespread, our study confirms that ASC is a neural systems disorder with neurodevelopmental differences in white matter brain connectivity.

## Supplementary information

**Additional file 1.** Supplementary figure. Results of region of interest analysis.

## Data Availability

The authors have full access to all the data supporting the conclusions of this article, which will be shared on an open access repository, and should the manuscript be accepted for publication.

## Abbreviations

ADI-R: Autism Diagnostic Interview-Revised
ADOS: Autism Diagnostic Observation Schedule
ASC: Autism spectrum condition
BET: Brain extraction tool
DTI: Diffusion tensor imaging
FA: Fractional anisotropy
FMRIB: Oxford Center for functional MRI of the brain
FSL: FMRIB Software Library
GLM: General linear model
ICD-10: International Classification of Diseases-10
IFOF: Inferior fronto-occipital fasciculus
ILF: Inferior longitudinal fasciculus
IOPPN: Institute of Psychiatry, Psychology & Neuroscience
IQ: Intelligence quotient
KCL: King’s College London
MD: Mean diffusivity
MRI: Magnetic resonance imaging
RD: Radial diffusivity
ROI: Region of interest
SLF: Superior longitudinal fasciculus
TBSS: Tract-based spatial statistics
UF: Uncinate fasciculus
UK: United Kingdom
WASI: Wechsler Abbreviated Scale of Intelligence
WM: White matter
