# Supplementary material for: Age-related differences in white matter diffusion measures in autism spectrum condition
Source: Mol Autism. 2020 May 18;11:36. doi: 10.1186/s13229-020-00325-6 (PMC7236504; doi:10.1186/s13229-020-00325-6)
Supplement: Supplementary file 1 — Additional file 1. Supplementary figure. Results of region of interest analysis. [file 13229_2020_325_MOESM1_ESM.docx]

**Supplementary Material**

**Supplementary Figure. Results of Region of Interest Analysis**

Scatter plots of raw data with superimposed mean linear development trajectories for sixteen white matter pathways. Blue corresponds to participants with ASC, and Red with typically developing controls.

**
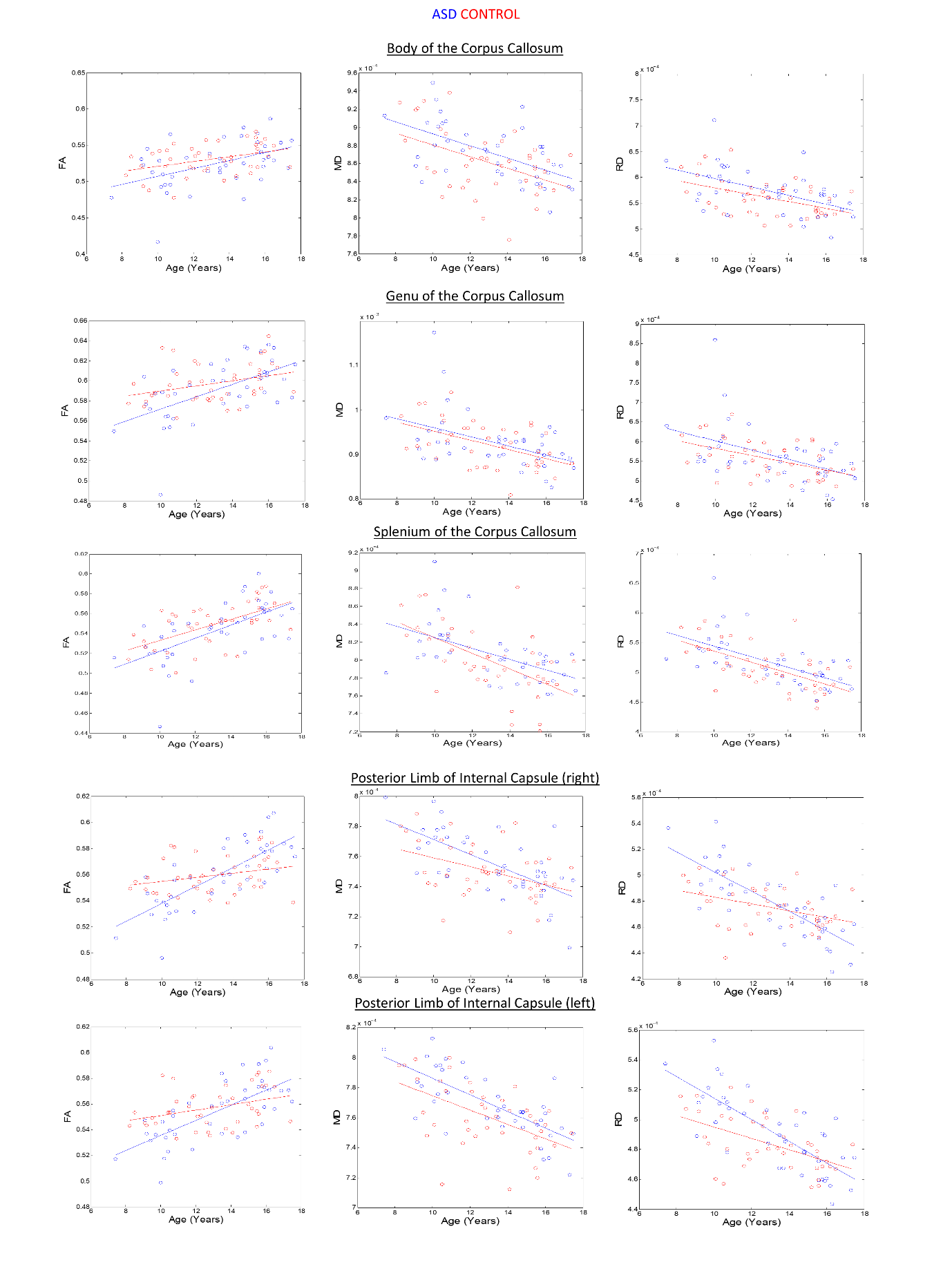
**


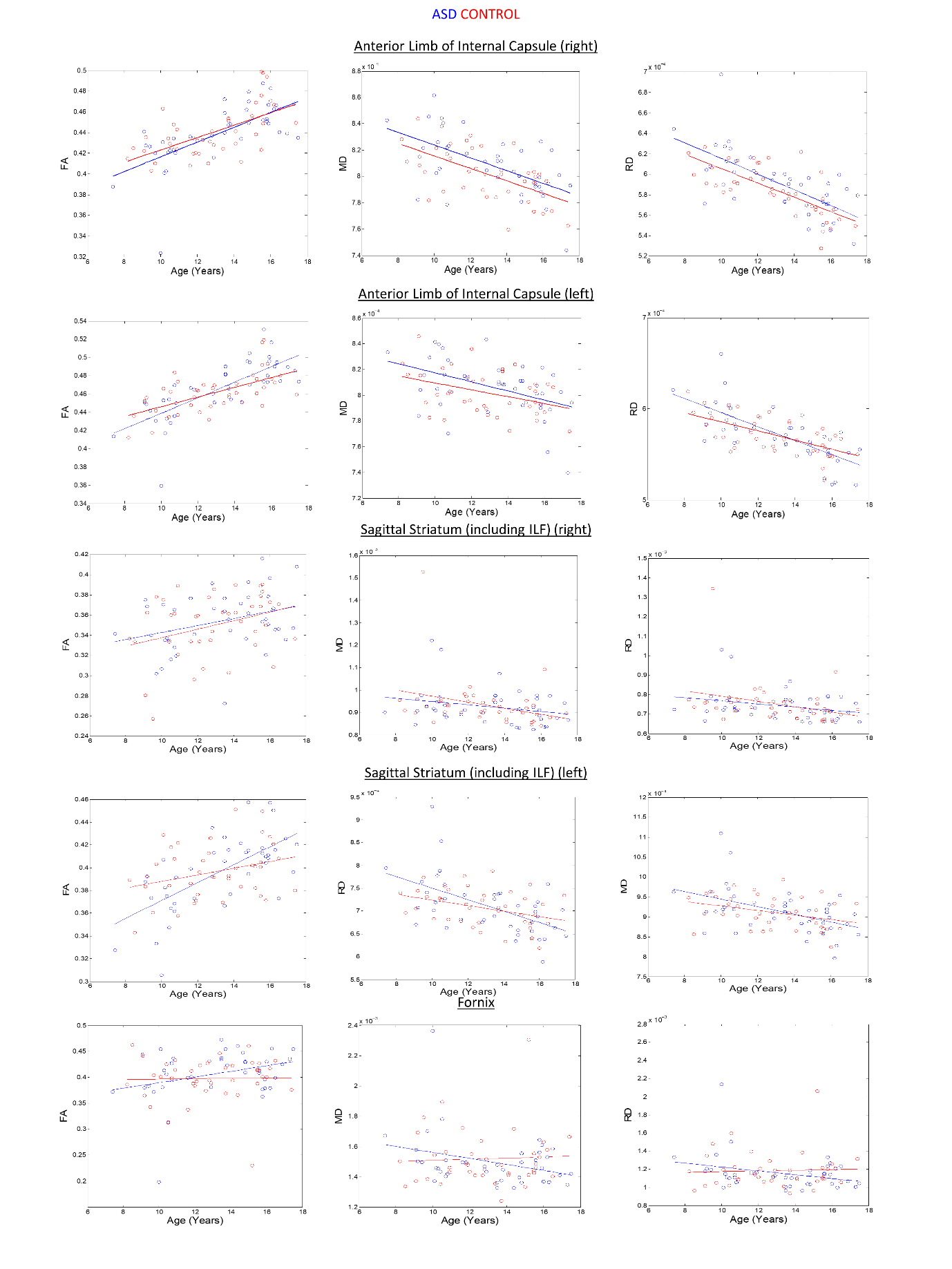


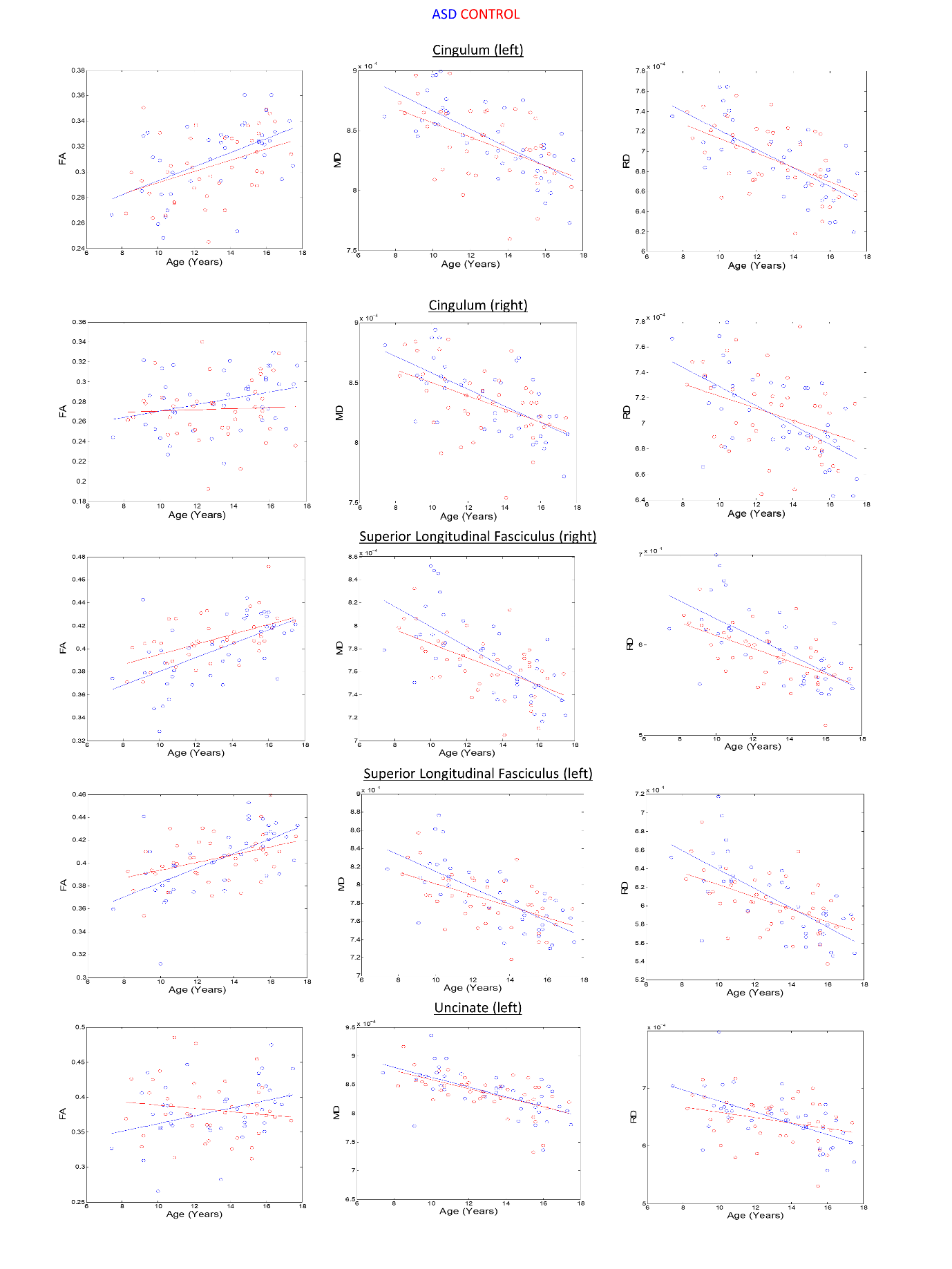


**
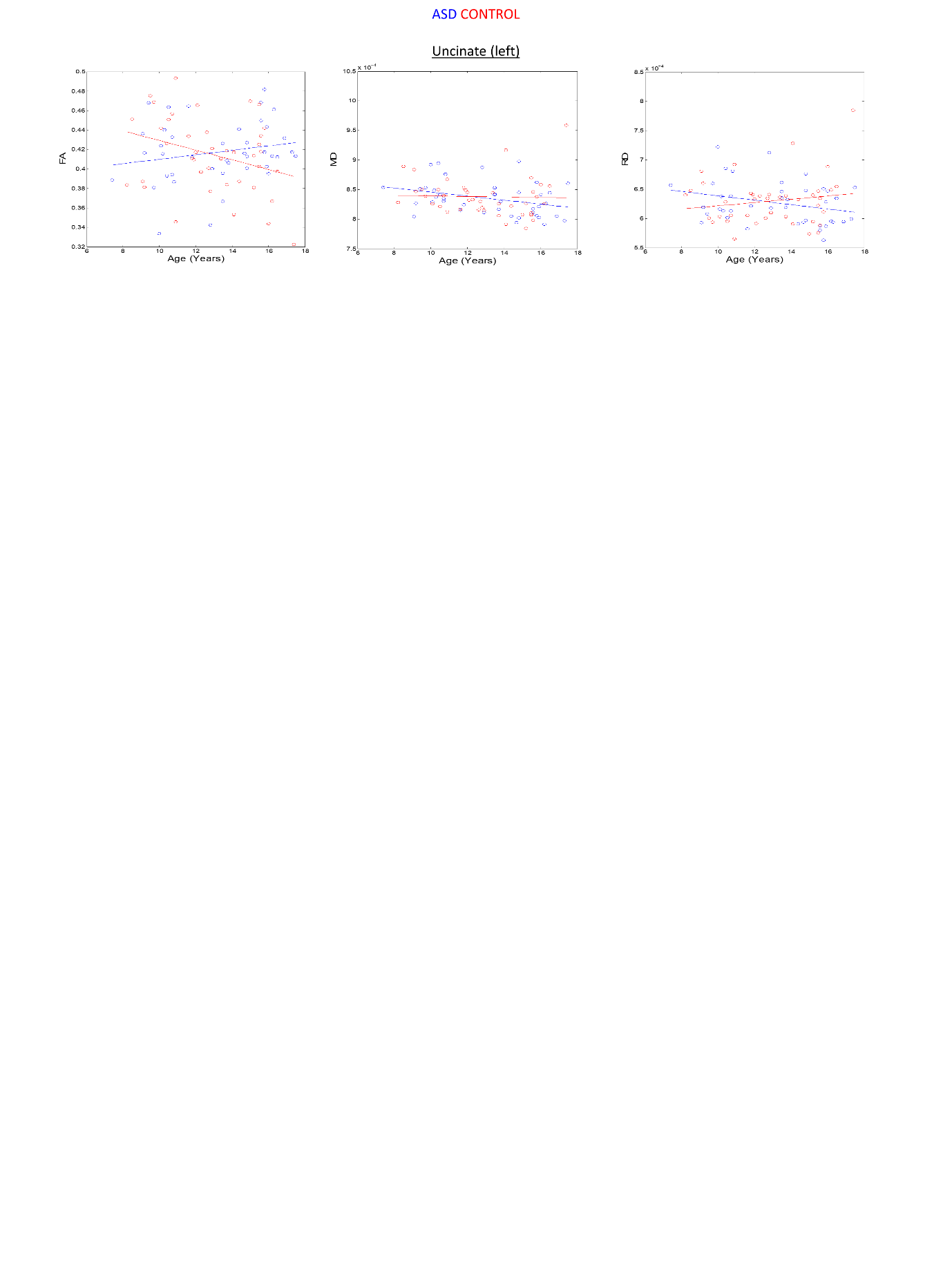
**
